# Economic Rationality and Health Behavior: Investigating the Link Between Financial Literacy and the BMI

**DOI:** 10.3390/bs15050632

**Published:** 2025-05-06

**Authors:** Kota Ogura, Honoka Nabeshima, Tomoka Kiba, Sakiho Aizawa, Hibiki Nagahama, Haruka Izumi, Mostafa Saidur Rahim Khan, Yoshihiko Kadoya

**Affiliations:** School of Economics, Hiroshima University, 1-2-1 Kagamiyama, Higashihiroshima 7398525, Japand254933@hiroshima-u.ac.jp (H.N.); ykadoya@hiroshima-u.ac.jp (Y.K.)

**Keywords:** BMI, financial literacy, obesity prevention, rational decision-making, health investment

## Abstract

Obesity is a major global health concern related to chronic diseases and rising healthcare costs. While previous studies focused on diet habits, environmental issues, and physical activity, financial literacy remains an overlooked factor in weight management. This study examined the relationship between financial literacy and the body mass index (BMI), using financial literacy as a proxy for rational health decision-making. A quantitative approach was employed, where linear regression analyzed the BMI as a continuous variable and a probit regression assessed overweight, normal weight, and underweight categories. A nationwide survey, the Preference Parameter Study, conducted by Osaka University, Japan, in the United States, provided the data for this study. The results indicate a significant negative association between financial literacy and the BMI, with higher financial literacy linked to a lower BMI and a greater likelihood of maintaining a normal weight. The key control variables, including impatience, gender, education, income, and smoking, also significantly affected the BMI. These findings reflect a strong correlation between financial literacy and the weight status; however, due to data limitations, causal inferences could not be made. We acknowledge the potential endogeneity and the cross-sectional nature of the data as limitations. Thus, while our results suggest a potential role for financial literacy in promoting rational health behavior, the policy implications should be interpreted with caution. Future research should explore targeted interventions across various demographic groups to maximize the impact.

## 1. Introduction

Obesity is a critical global health concern contributing to various chronic diseases, such as cardiovascular disease, type two diabetes, and hypertension (Guo et al., 2024; WHO, 2021; Fukawa, 2020; Yang et al., 2021; Hidese et al., 2018; Bastien et al., 2014). The rising prevalence of obesity has prompted researchers to explore its determinants, including lifestyle choices, socioeconomic status, and psychological factors (Benbaibeche et al., 2023; Kranjac & Kranjac, 2023; Mohd-Sidik et al., 2021; Al Yazeedi et al., 2020; Wilkie et al., 2016; Brockmann et al., 2017; Lloyd et al., 2014). However, existing efforts have yet to provide a comprehensive understanding and solution to this issue (Khattab, 2024). Although interventions focus primarily on diet habits, physical activity, and medical treatments, they often overlook the role of financial literacy in shaping health-related behavior (Benbaibeche et al., 2023; Kranjac & Kranjac, 2023; Mohd-Sidik et al., 2021). Therefore, understanding the BMI through the lens of financial literacy remains crucial, offering an alternative perspective on individuals’ ability to make rational decisions about their health, diet, and long-term well-being.

Obesity is considered as a health behavioral problem, shaped by individual lifestyle choices that provide short-term benefits but entail long-term costs. Ikeda et al. (2010) provided evidence that impatience and present bias significantly impact one’s BMI, suggesting that individuals may prioritize unhealthy diet habits and physical inactivity for immediate gratification, leading to long-term negative consequences, such as chronic diseases, reduced productivity, and increased healthcare costs. Obesity can be analyzed through the irrational choice framework, where cognitive biases, impulsivity, and an underestimate of long-term health risks contribute to overeating and a sedentary lifestyle (Ikeda et al., 2010; Barbour, 2023; Wang et al., 2021; van Sluijs et al., 2021). These behaviors reflect a present bias and imperfect information processing, preventing individuals from fully understanding the risks of obesity. A potential solution lies in adopting a rational choice framework grounded in Grossman’s human capital model (Grossman, 1972, 2000), which conceptualizes health as an investment. In this model, obesity is viewed as a failure to invest in health capital, leading to decreased productivity and well-being. Rational decision-making requires individuals to weigh the long-term costs of obesity against the short-term pleasures of unhealthy behaviors. This theoretical approach underscores the need for an empirical examination of how rational decision-making influences one’s BMI, helping individuals overcome cognitive limitations and biases, make informed health choices, reduce obesity, and improve overall health outcomes.

We used financial literacy as a proxy for rational decision-making to operationalize Grossman’s human capital model (Grossman, 1972, 2000) to explain obesity. Previous studies found that financially literate individuals are more likely to allocate resources efficiently, make long-term plans, and are less likely to experience cognitive biases, enabling them to make rational decisions, particularly in budgeting and investment (Hardika et al., 2024; Lusardi & Mitchell, 2014; Khan et al., 2020, 2021; Lusardi & Mitchell, 2011, 2014). Beyond financial outcomes, financial literacy has also been shown to promote positive health behaviors, such as regular exercise, reduced alcohol consumption, smoking cessation, and lower anxiety levels (Kadoya & Khan, 2017; Watanapongvanich et al., 2020, 2021). Just as individuals make strategic investments in education and career development, rational individuals also engage in economic decision-making regarding their health, including maintaining a balanced diet, participating in physical activity, and avoiding excessive calorie consumption (Barbour, 2023; Wang et al., 2021; van Sluijs et al., 2021). Thus, financially literate people are likely to recognize the long-term benefits of maintaining an optimal BMI and make informed choices that contribute to improved health outcomes.

Although financial literacy’s impact on other health-risk behaviors, such as smoking, a lack of exercise, and alcohol consumption, have been explored (Kadoya & Khan, 2017; Watanapongvanich et al., 2020, 2021), there remains a gap in empirical research examining its direct association with the BMI. Previous studies have focused primarily on health education, food environment, and lifestyle interventions but have not systematically examined financial literacy as a proxy for rational decision-making in obesity prevention (Benbaibeche et al., 2023; Kranjac & Kranjac, 2023; Al Yazeedi et al., 2020; Mohd-Sidik et al., 2021). Our study sought to address this gap by examining the association between financial literacy and the BMI, while explicitly acknowledging that the analysis was correlational and not causal. Due to the cross-sectional nature of the data and the lack of suitable instruments or longitudinal design, causal inference was not possible. However, we controlled for key confounders, such as education, impatience, and time preferences, to mitigate potential bias.

This study aimed to investigate the relationship between financial literacy and the BMI in the United States by examining whether individuals with a higher financial literacy tended to have lower levels of the BMI. We hypothesized that financial literacy may influence health decision-making processes, though we do not assert a direct causal relationship. We argue that financial literacy was associated with patterns in resource allocation, diet quality, and long-term health planning that were linked to weight outcomes. As a result, individuals with higher financial awareness may engage in more disciplined weight management and show less reliance on maladaptive coping strategies, such as stress-induced overeating.

This paper makes several contributions to the existing literature on health economics and behavioral finance. First, it offers a novel perspective on the obesity phenomenon by examining its association with financial literacy within the context of a rational decision-making framework. While we use financial literacy as a proxy for rational thinking, we acknowledge that the cross-sectional nature of the data limits any causal interpretation. Second, this paper highlights a potential link between financial literacy and health-related outcomes, suggesting that individuals with higher financial literacy may also be more likely to engage in behaviors associated with a healthier weight status. These findings contribute to the growing conversation around the broader relevance of financial education and provide a foundation for future research exploring its role in public health contexts.

## 2. Data and Methods

### 2.1. Data

This study utilized data from the Preference Parameters Study (PPS) conducted by the Institute of Social and Economic Research at Osaka University. The PPS is a panel survey conducted annually from 2005 to 2013 in the United States. For this study, we used data from the 2010 wave of the survey, which included information on height, weight, financial literacy, and socioeconomic characteristics and preferences. The respondents of the survey were from the District of Columbia and 48 other states (excluding Alaska and Hawaii), representing the population of the United States. The survey employed a multistage stratified random sampling method to select participants. A structured questionnaire was used to collect information, incorporating dichotomous, multiple-choice, and scaling questions on the demographic, socioeconomic, and psychological characteristics and preferences of the participants.

The 2010 wave of the survey was selected, as it included financial literacy measures, which were absent from the more recent waves. The dataset initially contained responses from 7046 participants. However, after excluding samples with missing values, the final analytical sample consisted of 3547 responses, which accounted for 50.34% of the total responses. To ensure the exclusion of missing data did not introduce bias, we compared the distribution of the excluded data with the retained sample and found no significant differences in the key demographic and socioeconomic variables. Therefore, the missing data exclusion was unlikely to affect the validity of our findings.

### 2.2. Variables

To investigate the impact of financial literacy on the body mass index (BMI), we categorized four dependent variables: BMI, overweight, normal range, and underweight. We first calculated the BMI using the descriptive question, “What are your height and weight?” The respondents provided their height in feet and inches with their weight in pounds, so the BMI was calculated using the following formula:

$$BMI=\frac{weight\left(Ib\right)}{{height(in)}^{2}}\times 703$$

Then, the BMI was classified into three categories following the World Health Organization (WHO) standards: the respondents with a BMI of more than 25 were considered overweight, those with a BMI between 18.5 and 25 were considered in the normal range, and those with a BMI below 18.5 were considered underweight.

Our primary independent variable, financial literacy, was constructed using Lusardi and Mitchell’s financial literacy measurement questions (Lusardi & Mitchell, 2008) (see Appendix A for details). These questions assess mathematical skills and the ability to understand fundamental financial concepts, such as interest rates, inflation, and risk diversification, which are essential for rational investment decisions. Due to their simplicity and adaptability, this method has been widely used in numerous studies to measure financial literacy (Watanapongvanich et al., 2020, 2021; Zheng et al., 2021). Additionally, Nicolini and Haupt (2019) highlight the practicality and reliability of these questions. Based on these considerations, we adopted this approach to quantify respondents’ financial literacy. Each correct answer was assigned 1 point, while incorrect answers received 0 points. Following previous studies (Watanapongvanich et al., 2020, 2021), we calculated the total score and normalized it to a scale of 0 to 1 to ensure comparability across the respondents.

Similar to relevant studies on health behavior (Watanapongvanich et al., 2020, 2021), our study included demographic, socioeconomic, behavioral, and perceptual control variables, such as risk aversion, sex, university graduation, age, income, work hours, and smoking behavior. Additionally, we controlled for impatience, hyperbolic discounting, and the sign effect based on the findings of Ikeda et al. (2010). These variables were constructed following the methodology of Ikeda et al. (2010) using five survey questions (see Appendix B for details). For example, Question 1 asks the respondents whether they prefer to receive a reward today or in 7 days. Typical individuals prefer immediate rewards (Option A) over delayed ones (Option B). However, when the utility of choosing Option B exceeds that of Option A, the respondents switch their choice to B. This switching continues as the interest rate progressively increases with each subsequent question. The respondents’ discount rates were calculated based on the interest rates at their switching points, denoted as DR1. The discount rates for Questions 2–5 were similarly calculated, which controlled for the reward size, time horizon, and receipt or payment terms, denoted as DR2–DR5^1^. The respondents who switched their choices multiple times were excluded from the analysis. Assuming a log-normal distribution for gross discount rates across the respondents, impatience was created as the mean of the standardized values of DR1 to DR5, as calculated by the following formula:

$$Impatience=\frac{1}{5}\sum_{i=1}^{5}\left[\left({DR}_{i}-E\left({DR}_{i}\right)\right)/\sigma ({DR}_{i})\right]$$

Hyperbolic discounting is identified when respondents exhibit a higher impatience over shorter time horizons than longer ones (DR1 > DR2). A sign effect is identified when respondents exhibit a higher impatience when receiving than when paying (DR4 > DR5). All variables used for our analysis and their descriptions are summarized in Table 1.

### 2.3. Methods

This study employed a quantitative research approach to examine the relationship between financial literacy and the BMI. Given that the BMI is a continuous variable, we used a linear regression analysis as our primary estimation model. Linear regression is appropriate for modeling relationships between continuous dependent variables and independent predictors, which allowed us to assess the direct impact of financial literacy on the BMI while controlling for potential confounders, such as age, income, education, and lifestyle factors.

To ensure the robustness of our findings, we employed alternative models that categorized the BMI into discrete groups: overweight, normal range, and underweight. Since these alternative dependent variables are binary in nature, we applied probit regression analysis to estimate their relationship with financial literacy. Probit regression is well-suited for modeling binary outcomes, as it accounts for the probability distribution of categorical dependent variables and ensures that predicted probabilities fall within a valid range (0 to 1).

The use of both linear and probit regression models provided a comprehensive understanding of how financial literacy influenced the BMI. Linear regression allowed for an interpretation of the marginal effects on the BMI as a continuous measure, while the probit regression enabled us to evaluate how financial literacy impacted the likelihood of the individuals being classified into the specific BMI categories. This dual approach strengthened the reliability of our findings and ensured that our conclusions remained valid across different analytical frameworks.

The primary equation for estimation was as follows:

$$Y_{i}=f({FL}_{i},X_{i},\varepsilon_{i}),$$

where Yi represents the BMI of the ith respondent or whether they fell into the categories of overweight (BMI ≥ 25), normal range (BMI ≥ 18.5 and BMI < 25), or underweight (BMI < 18.5). FL denotes the average financial literacy score, X represents a vector of respondent’s characteristics, and ε is the error term.

Since financial literacy may be strongly correlated with several other independent variables, such as hyperbolic discounting, having a university degree, and smoking behavior (Watanapongvanich et al., 2021), our results may face multicollinearity issues. Following Watanapongvanich et al. (2020, 2021), we conducted a variance inflation factor (VIF) test (available upon request) and found that multicollinearity was negligible (that is, the VIF values were below 10) for all the variables except for age and age squared across all the models.

The specifications of our model were as follows:

$$\begin{array}{cc} {BMI}_{i}= & \beta_{0}+\beta_{1}{Financial\;literacy}_{i}+\beta_{2}{Impatience}_{i}\\ & +\beta_{3}{Hyperbolic\;discounting}_{i}+\beta_{4}{Sign\;effect}_{i}\\ & +\beta_{5}{Risk\;aversion}_{i}+\beta_{6}{Male}_{i}+\beta_{7}{University\;degree}_{i}\\ & +\beta_{8}{Age}_{i}+\beta_{9}{Age\;squared}_{i}+\beta_{10}{Income\;squared}_{i}\\ & +\beta_{11}{Workhour\;square\;root}_{i}+\beta_{12}{Smoking}_{i}+\varepsilon_{i} \end{array}$$

$$\begin{array}{cc} {Overweight}_{i}= & \beta_{0}+\beta_{1}{Financial\;literacy}_{i}+\beta_{2}{Impatience}_{i}\\ & +\beta_{3}{Hyperbolic\;discounting}_{i}+\beta_{4}{Sign\;effect}_{i}\\ & +\beta_{5}{Risk\;aversion}_{i}+\beta_{6}{Male}_{i}+\beta_{7}{University\;degree}_{i}\\ & +\beta_{8}{Age}_{i}+\beta_{9}{Age\;squared}_{i}+\beta_{10}{Income\;squared}_{i}\\ & +\beta_{11}{Workhour\;square\;root}_{i}+\beta_{12}{Smoking}_{i}+\varepsilon_{i} \end{array}$$

$$\begin{array}{cc} {Normal\;range}_{i}= & \beta_{0}+\beta_{1}{Financial\;literacy}_{i}+\beta_{2}{Impatience}_{i}\\ & +\beta_{3}{Hyperbolic\;discounting}_{i}+\beta_{4}{Sign\;effect}_{i}\\ & +\beta_{5}{Risk\;aversion}_{i}+\beta_{6}{Male}_{i}+\beta_{7}{University\;degree}_{i}\\ & +\beta_{8}{Age}_{i}+\beta_{9}{Age\;squared}_{i}+\beta_{10}{Income\;squared}_{i}\\ & +\beta_{11}{Workhour\;square\;root}_{i}+\beta_{12}{Smoking}_{i}+\varepsilon_{i} \end{array}$$

$$\begin{array}{cc} {Underweight}_{i}= & \beta_{0}+\beta_{1}{Financial\;literacy}_{i}+\beta_{2}{Impatience}_{i}\\ & +\beta_{3}{Hyperbolic\;discounting}_{i}+\beta_{4}{Sign\;effect}_{i}\\ & +\beta_{5}{Risk\;aversion}_{i}+\beta_{6}{Male}_{i}+\beta_{7}{University\;degree}_{i}\\ & +\beta_{8}{Age}_{i}+\beta_{9}{Age\;squared}_{i}+\beta_{10}{Income\;squared}_{i}\\ & +\beta_{11}{Workhour\;square\;root}_{i}+\beta_{12}{Smoking}_{i}+\varepsilon_{i} \end{array}$$

## 3. Results

### 3.1. Descriptive Statistics

As illustrated in Table 2, the average BMI of the respondents was 28.19. The proportions of each variable related to the BMI among all the respondents were as follows: 64.14% were overweight (BMI ≥ 25), 34.14% were in the normal range (18.5 ≥ BMI and BMI < 25), and 1.72% were underweight (BMI < 18.5). The average financial literacy score of the respondents was 0.71. The mean value of impatience, which represents the standardized value of the time discount rate of the respondents from DR1 to DR5, was 1.52 × 10^−10^ (see Table 3 for the detailed descriptive statistics of DR1 to DR5). The proportions of respondents who displayed hyperbolic discounting and the sign effect were 10.83% and 65.52%, respectively. On average, they would take an umbrella with them if the chance of rain was 66.83%. We also found that 46.88% were male, and 43.14% obtained a university degree. The average respondents were middle-aged people around 49 years old. Furthermore, the respondents had an average annual household income of around USD 69,000. The respondents’ average weekly working hours were around 25 h. Finally, we found that many respondents did not have smoking habits.

### 3.2. Regression Results

Table 4 shows the estimation results for the relationship between the financial literacy and the BMI, including robustness checks using alternative models that categorized the BMI into the overweight, normal range, and underweight groups. The primary analysis using the BMI as a continuous dependent variable revealed a significant negative association between financial literacy and the BMI (−1.1368, *p* < 0.01), indicating that individuals with a higher financial literacy tended to have lower BMI levels, supporting the hypothesis that rational decision-making, facilitated by financial literacy, contributed to healthier weight management. The robustness of this finding was confirmed through alternative model specifications that categorized the BMI into the overweight, normal range, and underweight groups.

In the probit regression models, financial literacy remained a significant predictor in all the BMI categories. A higher financial literacy was associated with a lower probability of being overweight (−0.2086, *p* < 0.01) and a higher probability of maintaining a normal BMI (0.2442, *p* < 0.01). However, financial literacy did not show a statistically significant relationship with the probability of being underweight (−0.2632, *p* > 0.1), suggesting that its influence primarily supported the maintenance of a healthy weight rather than contributing to the underweight status.

Among the control variables, impatience was positively correlated with the BMI (0.5775, *p* < 0.01), indicating that individuals with a higher impatience were more likely to have a higher BMI. Male respondents tended to have significantly higher BMI levels (0.9875, *p* < 0.01) and were more likely to be overweight, while females were more likely to fall within the normal weight range. A university degree was associated with a lower BMI (−1.2238, *p* < 0.01), a reduced likelihood of being overweight (−0.2114, *p* < 0.01), and an increased probability of normal weight classification (0.1924, *p* < 0.01).

Age followed a non-linear relationship with the BMI, as evidenced by the positive coefficient for age (0.4511, *p* < 0.01) and the negative coefficient for age squared (−0.0043, *p* < 0.01). This suggests that the BMI increased with age but at a decreasing rate. A similar trend was observed for income squared, where higher income levels correlated with a lower BMI, probably due to an increased access to healthier lifestyle options. Work hours also exhibited a small but significant negative association with the BMI (−0.0990, *p* < 0.05), implying that increased work commitments might contribute to better weight management.

Smoking was significantly negatively associated with the BMI (−0.5869, *p* < 0.01) and reduced the probability of being overweight, while it increased the likelihood of being in the normal or underweight categories.

## 4. Discussion

Our findings provide evidence of a significant negative association between financial literacy and the BMI, supporting the rational decision-making framework proposed in this study. The negative association between financial literacy and the BMI aligns with previous research suggesting that individuals with higher financial literacy are more likely to make informed health-related choices, invest in nutritious foods, and resist the temptation of immediate gratification from unhealthy eating habits (Lusardi & Mitchell, 2014; Hardika et al., 2024). These results reinforce the notion that financially literate individuals approach health decisions similarly to financial investments, emphasizing long-term benefits over short-term indulgences. Furthermore, the robustness checks using probit models confirmed that financial literacy significantly reduced the likelihood of being overweight while increasing the probability of maintaining a normal BMI. However, the lack of a significant relationship with underweight status suggests that financial literacy primarily influenced weight management rather than contributed to extreme weight loss behaviors.

The association of financial literacy with the BMI also aligns with previous studies on rational health behaviors, where financially literate individuals demonstrate better control over habits related to smoking, alcohol consumption, and physical activity (Kadoya & Khan, 2017; Watanapongvanich et al., 2020, 2021). This study expanded on this literature by establishing a direct connection between financial literacy and weight management, reinforcing the argument that economic knowledge improves rational health investments. Unlike studies that focused primarily on health education, lifestyle, or environmental factors (Khattab, 2024; Wiechert & Holzapfel, 2021; Gato-Moreno et al., 2021; Huang et al., 2023), our findings suggest that financial literacy could play a complementary role in obesity prevention by supporting long-term health planning and informed lifestyle choices. However, we emphasize that our findings reflect correlations, not causal relationships.

While the findings provide valuable insights into the behavioral link between financial literacy and the BMI, it is important to consider the temporal context of the dataset. The data used in this study were collected between 2005 and 2013, prior to major societal shifts driven by technological advancements, digital finance tools, and the rise of AI. These developments may have influenced both financial behaviors and lifestyle patterns in recent years, potentially affecting the strength or nature of the observed relationships. Although the core cognitive and behavioral mechanisms underlying financial literacy are relatively stable, future studies using more recent data are essential to assess the continued relevance and generalizability of our findings in today’s dynamic environment.

Regarding the control variables, impatience was found to be positively correlated with the BMI, implying that individuals who prioritize short-term gratification over long-term well-being are more likely to experience weight gain. This is consistent with the literature on behavioral economics (Ikeda et al., 2010), where impatience has been linked to poor diet habits and lower levels of physical activity (Pastore et al., 2023; Courtemanche et al., 2015). Similarly, the significant effect of male gender on a higher BMI aligns with existing evidence that the causes of obesity for men could be attributed to dietary differences compared with women, where women exhibit a stronger preference for dietary foods (Feraco et al., 2024; Wardle et al., 2004).

Educational attainment, measured by the status of a university degree, was associated with a lower BMI and a higher likelihood of being in the normal weight range. This supports previous findings stating that a higher education is related to better health awareness and access to healthier food options (Witkam et al., 2021; Cohen et al., 2013; Mazzocchi et al., 2009). The observed non-linear relationship between age and the BMI, as indicated by the positive coefficient for age and the negative coefficient for age squared, confirmed the widely accepted trend that the BMI increases with age but at a decreasing rate.

Additionally, income and work hours were found to have a modest but significant effect on the BMI. Higher income levels correlated with a lower BMI, probably due to increased access to healthier lifestyle options and healthcare resources. Conversely, individuals with longer working hours tended to have a lower BMI, suggesting that increased time commitments might reduce the opportunities for excessive eating or sedentary behaviors. These findings are consistent with previous studies that examined socioeconomic influences on weight management (Safaei et al., 2021; Chatterjee et al., 2020).

Smoking is another key determinant, and our results show a strong negative association between smoking and the BMI. It is well established that obesity rates tend to be lower among smokers compared with non-smokers (Bamia et al., 2004). However, several studies, including those by Canoy et al. (2005) and Czernichow et al. (2004), have found higher obesity rates among current smokers. Differences in sample characteristics, such as socioeconomic status, physical activity levels, and coexisting health conditions, could influence this relationship. For example, lower-income populations, which may have higher smoking rates, often have limited access to healthier food options and exercise opportunities, which could contribute to obesity. This may help explain the discrepancy between our findings and those of previous studies.

Despite its contributions, this study had certain limitations. First, the dataset used spans from 2005 to 2013, which may raise concerns about data obsolescence. Although this is the most recent dataset that includes both detailed financial literacy measures and an objectively measured BMI, the time gap could introduce a potential bias. However, we believe that this limitation had a minimal impact on this study’s findings, as the behavioral and cognitive mechanisms linking financial literacy with health-related choices tend to be relatively stable over time. Second, the temporal context of the data may limit the generalizability. Since the data were collected between 2005 and 2013, societal and technological changes, such as the rise of digital finance and AI, may have influenced financial behaviors and lifestyle choices. Third, although we attempted to control for relevant confounding variables, such as education, impatience, and time preferences, endogeneity remains a concern. Unobserved factors, such as personality traits, self-discipline, cognitive ability, or genetic predispositions, may simultaneously influence both financial literacy and the BMI. Moreover, reverse causality cannot be ruled out, as better physical and mental health associated with a lower BMI may also enhance an individual’s capacity to manage finances. Given these limitations, our findings should be interpreted as correlational rather than causal. Finally, this study was limited to data from the United States, which may restrict the generalizability of the findings for other countries with different cultural, economic, or health-related behaviors. This focus was primarily due to the lack of comparable international datasets that include both detailed financial literacy measures and an objectively measured BMI. Future research should utilize contemporary international datasets and adopt methods such as longitudinal designs, instrumental variables, or experiments to address endogeneity and more accurately assess the causal relationship between financial literacy and health outcomes.

## 5. Conclusions

This study provided empirical evidence for a significant association between financial literacy and the BMI, supporting the hypothesis that financially literate individuals are more likely to engage in healthier lifestyle choices. The negative association between financial literacy and the BMI suggests that financial knowledge enhances individuals’ ability to allocate resources effectively, invest in long-term health, and resist short-term gratification from unhealthy diet habits. While our findings are consistent with the rational decision-making framework, we emphasize that they reflect a correlation, not causation. Due to the cross-sectional nature of the data and potential endogeneity, causal inferences cannot be drawn. Nonetheless, the results reinforce the argument that financial literacy may extend beyond economic behavior and be relevant in the context of health-related decisions. Additionally, key control variables, such as impatience, gender, education, income, and smoking, all demonstrated significant associations with the BMI, highlighting the multifaceted nature of weight management. This study contributed to the literature by linking financial literacy to health outcomes, offering a novel behavioral perspective on obesity and highlighting the broader role of financial education in promoting healthier lifestyle choices.

Given the correlational nature of the findings, the policy implications should be interpreted with caution. However, the results suggest that financial literacy could be a valuable dimension to consider in the design of public health initiatives. Policymakers may explore the potential benefits of integrating financial literacy content into broader health promotion strategies. For example, financial education components could be included in school curricula, workplace wellness programs, or community outreach initiatives aimed at fostering long-term health planning and self-discipline. Future research using longitudinal data or experimental designs is essential to examine the causal pathways between financial literacy and health outcomes more rigorously. Interdisciplinary collaboration between economists, behavioral scientists, and healthcare professionals will be vital in developing integrated, evidence-based interventions. One promising direction could involve behavioral tools, such as commitment contracts, where individuals set health goals with financial incentives and accountability mechanisms to promote sustained behavioral change.

## Figures and Tables

**Table 1:** Variable definitions.

Variables	Definitions
BMI	Continuous variable: body mass index, defined as weight in pounds divided by height in inches squared and then multiplied by 703 $\left(\frac{lb}{{in}^{2}}\times 703\right)$
Overweight	Binary variable: 1—BMI ≥ 25 and 0—otherwise
Normal range	Binary variable: 1—BMI ≥ 18.5 and BMI < 25, and 0—otherwise
Underweight	Binary variable: 1—BMI < 18.5 and 0—otherwise
Financial literacy	Continuous variable: average score of Lusardi and Mitchell’s financial literacy measurement questions (Appendix A)
Impatience	Continuous variable: simple mean of the standardized values of the elicited discount rates DRi (i = 1, …, 5) as a measure of the degree of impatience
Hyperbolic discounting	Binary variable: 1—DR1 > DR2 and 0—otherwise
Sign effect	Binary variable: 1—DR4 > DR5 and 0—otherwise
Risk aversion	Continuous variable: percentage score, which is constructed by subtracting from 100 the respondent’s response to the question: “When you usually go out, how high does the probability of rain have to be before you take umbrella?”
Male	Binary variable: 1—male and 0—female
University degree	Binary variable: 1—obtained a bachelor’s degree (4 years) or some post-graduate studies and 0—otherwise
Age	Continuous variable: respondent’s age
Age squared	Continuous variable: age squared
Household income	Continuous variable: annual earned income before taxes and with bonuses of the entire household in 2009 (unit: USD)
Income squared	Continuous variable: income squared
Work hours	Continuous variable: work hours for a week
Work hours square root	Continuous variable: work hours square root
Smoking	Discrete variable: the strength of smoking habits on a 6-point scale, from 1 (do not smoke at all or quit) to 6 (smoking more than two packages of cigarettes a day)

**Table 2:** Descriptive statistics.

Variable	Mean	Standard Deviation (SD)	Min	Max
BMI	28.19	7.25	12.48	170.03
Overweight	0.6414	0.4797	0	1
Normal range	0.3414	0.4743	0	1
Underweight	0.0172	0.1300	0	1
Financial literacy	0.7140	0.3081	0	1
Impatience	1.52 × 10^−10^	0.6258	−0.7854	3.5524
Hyperbolic discounting	0.1083	0.3108	0	1
Sign effect	0.6552	0.4754	0	1
Risk aversion	0.3317	0.2783	0.01	1
Male	0.4688	0.4991	0	1
University degree	0.4314	0.4953	0	1
Age	48.87	16.12	18	96
Household income	69,254.3	49,543.47	5000	210,000
Work hours	24.93	21.57	0	100
Smoking	1.42	1.11	1	6
Observations	3547			

**Table 3:** Time discount rates under controlled conditions.

		DR1	DR2	DR3	DR4	DR5	Impatience
Choice condition	Timings (A) or (B)	Today or 7 days	90 days or 97 days	1 month or 13 months	1 month or 13 months	1 month or 13 months	-
	Amount for (A)	USD 100	USD 100	USD 100	USD 10,000	USD 10,000	-
	Receipt or payment	Receipt	Receipt	Receipt	Receipt	Payment	-
Descriptive statistics	Mean	4.01	6.54	0.3191	0.0850	0.1331	1.51 × 10^−10^
	Median	0.40	1.00	0.20	0.06	0.01	
	Std. dev.	9.90	13.85	0.4049	0.1702	0.2180	0.6258
	Obs.	3547	3547	3547	3547	3547	3547
Time discounting properties (*p*-value)		Hyperbolic discounting: DR1 > DR2			Sign effect: DR4 > DR5		

**Table 4:** Regression estimates for the relationship between the financial literacy and BMI categories.

	BMI	Overweight	Normal Range	Underweight
Financial literacy	−1.1368 ***	−0.2086 ***	0.2442 ***	−0.2632
	(0.4166)	(0.0785)	(0.0789)	(0.1805)
Impatience	0.5775 ***	0.0360	−0.0201	−0.1775
	(0.1908)	(0.0360)	(0.0361)	(0.1107)
Hyperbolic discounting	−0.0885	0.0456	−0.0206	−0.2566
	(0.3799)	(0.0715)	(0.0716)	(0.2068)
Sign effect	−0.3584	−0.0498	0.0652	−0.1365
	(0.2547)	(0.0479)	(0.0481)	(0.1142)
Risk aversion	−0.4475	−0.0502	0.0298	0.1813
	(0.4291)	(0.0804)	(0.0808)	(0.1896)
Male	0.9875 ***	0.4059 ***	−0.3876 ***	−0.2113 *
	(0.2434)	(0.0460)	(0.0461)	(0.1155)
University degree	−1.2238 ***	−0.2114 ***	0.1924 ***	0.2088 *
	(0.2607)	(0.0487)	(0.0489)	(0.1200)
Age	0.4511 ***	0.0739 ***	−0.0637 ***	−0.0766 ***
	(0.0407)	(0.0076)	(0.0076)	(0.0156)
Age squared	−0.0043 ***	−0.0006 ***	0.0005 ***	0.0007 ***
	(0.0004)	(0.0001)	(0.0001)	(0.0002)
Income squared	−0.0000 ***	−0.0000 ***	0.0000 ***	0.0000
	(0.0000)	(0.0000)	(0.0000)	(0.0000)
Work hour square root	−0.0990 **	0.0084	−0.0050	−0.0344 *
	(0.0441)	(0.0083)	(0.0083)	(0.0201)
Smoking	−0.5869 ***	−0.0805 ***	0.0710 ***	0.0907 **
	(0.1094)	(0.0202)	(0.0203)	(0.0447)
Constant	20.7553 ***	−1.2535 ***	0.9352 ***	−0.1757
	(0.9679)	(0.1782)	(0.1781)	(0.3544)
Observations	3547	3547	3547	3547
R-squared	0.0660			
Adjusted R-squared	0.0628	0.0556	0.0484	0.0816
Log likelihood	−11,936	−2186	−2167	−283.1
F-value	20.79			
*p*-value	0.00	0.00	0.00	5.45 × 10^−7^

Notes: Robust standard errors in parentheses. *** *p* < 0.01, ** *p* < 0.05, * *p* < 0.1.

## Data Availability

The original data presented in this paper are openly available in the Institute of Social and Economic Research, Osaka University, at https://www.iser.osaka-u.ac.jp/survey_data/eng_application.html (accessed on 20 February 2025).

## Note

1 Contrary to Questions 1–4 assuming receipt situations, Question 5 assumes a payment situation to elicit what maximum amount respondents would prefer for a 12-month late payment. Typical individuals favor delayed payments (Option B) over immediate ones (Option A). However, when the utility of choosing Option A exceeds that of Option B, respondents switch their choice to A. This switching continues as the interest rate progressively increases with each subsequent question. We calculated the discount rate with an interest rate at their switching points and denoted it as DR5.

## Appendix A

Suppose you had $100 in a savings account and the interest rate is 2% per year and you never withdraw money or interest payments. After 5 years, how much would you have in this account in total?

- More than $102
- Exactly $102
- Less than $102
- Do not know
- Refuse to answer

Imagine that the interest rate on your savings account was 1% per year and the inflation was 2% per year. After 1 year, how much would you be able to buy with the money in this account?

- More than today
- Exactly the same
- Less than today
- Do not know
- Refuse to answer

Please indicate whether the following statement is true or false. “Buying a company stock usually provides a safer return than a stock mutual fund.”

- True
- False
- Do not know
- Refuse to answer

## Appendix B

**Table A1:** Q1. Let’s assume you have two options to receive some money. You may choose Option A, to receive $100 today; or Option B, to receive a different amount in seven days. Compare the amounts and timing in Option A with Option B and indicate which amount you would prefer to receive for all 9 choices.

	Option A	Option B	Annual Interest Rate
(1)	Receive USD 100 today	Receive USD 99.81 in 7 days	−10%
(2)	Receive USD 100 today	Receive USD 100 in 7 days	0%
(3)	Receive USD 100 today	Receive USD 100.19 in 7 days	10%
(4)	Receive USD 100 today	Receive USD 100.76 in 7 days	40%
(5)	Receive USD 100 today	Receive USD 101.91 in 7 days	100%
(6)	Receive USD 100 today	Receive USD 103.83 in 7 days	200%
(7)	Receive USD 100 today	Receive USD 105.74 in 7 days	300%
(8)	Receive USD 100 today	Receive USD 119.17 in 7 days	1000%
(9)	Receive USD 100 today	Receive USD 195.89 in 7 days	5000%

**Table A2:** Q2. Now let’s assume that you have the option to receive $100 in ninety days or receive a different amount in ninety-seven days. Compare the amounts and timing in Option A with Option B and indicate which amount you would prefer to receive for all 9 choices.

	Option A	Option B	Annual Interest Rate
(1)	Receive USD 100 in 90 days	Receive USD 99.81 in 97 days	−10%
(2)	Receive USD 100 in 90 days	Receive USD 100 in 97 days	0%
(3)	Receive USD 100 in 90 days	Receive USD 100.19 in 97 days	10%
(4)	Receive USD 100 in 90 days	Receive USD 100.76 in 97 days	40%
(5)	Receive USD 100 in 90 days	Receive USD 101.91 in 97 days	100%
(6)	Receive USD 100 in 90 days	Receive USD 103.83 in 97 days	200%
(7)	Receive USD 100 in 90 days	Receive USD 105.74 in 97 days	300%
(8)	Receive USD 100 in 90 days	Receive USD 119.17 in 97 days	1000%
(9)	Receive USD 100 in 90 days	Receive USD 195.89 in 97 days	5000%

**Table A3:** Q3. Now let’s assume that you have the option to receive $100 in one month or receive a different amount in thirteen months. Compare the amounts and timing in Option A with Option B and indicate which amount you would prefer to receive for all 10 choices.

	Option A	Option B	Annual Interest Rate
(1)	Receive USD 100 in 1 month	Receive USD 95 in 13 months	−5%
(2)	Receive USD 100 in 1 month	Receive USD 100 in 13 months	0%
(3)	Receive USD 100 in 1 month	Receive USD 102 in 13 months	2%
(4)	Receive USD 100 in 1 month	Receive USD 104 in 13 months	4%
(5)	Receive USD 100 in 1 month	Receive USD 106 in 13 months	6%
(6)	Receive USD 100 in 1 month	Receive USD 110 in 13 months	10%
(7)	Receive USD 100 in 1 month	Receive USD 120 in 13 months	20%
(8)	Receive USD 100 in 1 month	Receive USD 140 in 13 months	40%
(9)	Receive USD 100 in 1 month	Receive USD 180 in 13 months	80%
(10)	Receive USD 100 in 1 month	Receive USD 250 in 13 months	150%

**Table A4:** Q4. Now let’s assume that you have the option to receive $10,000 in one month or receive a different amount in thirteen months. Compare the amounts and timing in Option A with Option B and indicate which amount you would prefer to receive for all 10 choices.

	Option A	Option B	Annual Interest Rate
(1)	Receive USD 10,000 in 1 month	Receive USD 9500 in 13 months	−5%
(2)	Receive USD 10,000 in 1 month	Receive USD 10,000 in 13 months	0%
(3)	Receive USD 10,000 in 1 month	Receive USD 10,010 in 13 months	0.1%
(4)	Receive USD 10,000 in 1 month	Receive USD 10,050 in 13 months	0.5%
(5)	Receive USD 10,000 in 1 month	Receive USD 10,100 in 13 months	1%
(6)	Receive USD 10,000 in 1 month	Receive USD 10,200 in 13 months	2%
(7)	Receive USD 10,000 in 1 month	Receive USD 10,600 in 13 months	6%
(8)	Receive USD 10,000 in 1 month	Receive USD 11,000 in 13 months	10%
(9)	Receive USD 10,000 in 1 month	Receive USD 13,000 in 13 months	30%
(10)	Receive USD 10,000 in 1 month	Receive USD 20,000 in 13 months	100%

**Table A5:** Q5. Now let’s assume that you have the option to pay $10,000 in one month or pay a different amount in thirteen months. Compare the amounts and timing in Option A with Option B and indicate which amount you would prefer to pay for all 11 choices.

	Option A	Option B	Annual Interest Rate
(1)	Pay USD 10,000 in 1 month	Pay USD 8000 in 13 months	−20%
(2)	Pay USD 10,000 in 1 month	Pay USD 9000 in 13 months	−10%
(3)	Pay USD 10,000 in 1 month	Pay USD 9500 in 13 months	−5%
(4)	Pay USD 10,000 in 1 month	Pay USD 10,000 in 13 months	0%
(5)	Pay USD 10,000 in 1 month	Pay USD 10,010 in 13 months	0.1%
(6)	Pay USD 10,000 in 1 month	Pay USD 10,050 in 13 months	0.5%
(7)	Pay USD 10,000 in 1 month	Pay USD 10,100 in 13 months	1%
(8)	Pay USD 10,000 in 1 month	Pay USD 10,200 in 13 months	2%
(9)	Pay USD 10,000 in 1 month	Pay USD 10,600 in 13 months	6%
(10)	Pay USD 10,000 in 1 month	Pay USD 11,000 in 13 months	10%
(11)	Pay USD 10,000 in 1 month	Pay USD 15,000 in 13 months	50%
